# Preterm birth and structural brain alterations in early adulthood

**DOI:** 10.1016/j.nicl.2014.08.005

**Published:** 2014-08-13

**Authors:** Chiara Nosarti, Kie Woo Nam, Muriel Walshe, Robin M. Murray, Marion Cuddy, Larry Rifkin, Matthew P.G. Allin

**Affiliations:** a Department of Psychosis Studies, Institute of Psychiatry, King's Health Partners, King's College London, De Crespigny Park, SE58AF London, UK

**Keywords:** Very preterm, White matter, Grey matter, Brain volume, Cognitive outcome

## Abstract

Alterations in cortical development and impaired neurodevelopmental outcomes have been described following very preterm (VPT) birth in childhood and adolescence, but only a few studies to date have investigated grey matter (GM) and white matter (WM) maturation in VPT samples in early adult life. Using voxel-based morphometry (VBM) we studied regional GM and WM volumes in 68 VPT-born individuals (mean gestational age 30 weeks) and 43 term-born controls aged 19–20 years, and their association with cognitive outcomes (Hayling Sentence Completion Test, Controlled Oral Word Association Test, Visual Reproduction test of the Wechsler Memory Scale-Revised) and gestational age. Structural MRI data were obtained with a 1.5 Tesla system and analysed using the VBM8 toolbox in SPM8 with a customized study-specific template. Similarly to results obtained at adolescent assessment, VPT young adults compared to controls demonstrated reduced GM volume in temporal, frontal, insular and occipital areas, thalamus, caudate nucleus and putamen. Increases in GM volume were noted in medial/anterior frontal gyrus. Smaller subcortical WM volume in the VPT group was observed in temporal, parietal and frontal regions, and in a cluster centred on posterior corpus callosum/thalamus/fornix. Larger subcortical WM volume was found predominantly in posterior brain regions, in areas beneath the parahippocampal and occipital gyri and in cerebellum. Gestational age was associated with GM and WM volumes in areas where VPT individuals demonstrated GM and WM volumetric alterations, especially in temporal, parietal and occipital regions. VPT participants scored lower than controls on measures of IQ, executive function and non-verbal memory. When investigating GM and WM alterations and cognitive outcome scores, subcortical WM volume in an area beneath the left inferior frontal gyrus accounted for 14% of the variance of full-scale IQ (*F* = 12.9, *p* < 0.0001). WM volume in posterior corpus callosum/thalamus/fornix and GM volume in temporal gyri bilaterally, accounted for 21% of the variance of executive function (*F* = 9.9, *p* < 0.0001) and WM in the posterior corpus callosum/thalamus/fornix alone accounted for 17% of the variance of total non-verbal memory scores (*F* = 9.9, *p* < 0.0001). These results reveal that VPT birth continues to be associated with altered structural brain anatomy in early adult life, although it remains to be ascertained whether these changes reflect neurodevelopmental delays or long lasting structural alterations due to prematurity. GM and WM alterations correlate with length of gestation and mediate cognitive outcome.

## Highlights

• Preterm birth is associated with brain alterations in early adulthood
• Preterm birth affects maturation of both white and grey matter
• Volume alterations are observed in temporal, frontal, parietal and occipital areas
• Regional alterations mediate the effects of preterm birth on cognitive functioning

## 1 Introduction

Due to its rapidly developing and complex characteristics, the preterm brain is vulnerable to exogenous and endogenous insults in the third trimester of gestation (Volpe, 2009), during which the volume of the whole brain more than doubles and the volume of cortical grey matter (GM) increases approximately four-fold (Huppi et al., 1998). Therefore, attention has increasingly focused on the quality of life of survivors, who are at greater risk of brain damage and consequent neurological disorders, neuropsychological, and behavioural impairments in childhood and later in life (Ball et al., 2013; Beauchamp et al., 2008; Bjuland et al., 2013; Johnson and Marlow, 2011; Ment et al., 2009; Pavlova and Krageloh-Mann, 2013; Taylor et al., 2011).

Long-lasting and widespread alterations in brain structure in their second decade of life have been reported in individuals who were born very preterm (VPT; <32 weeks of gestation) and/or with a very low birth weight (VLBW; <1500 g). Volume reductions have been described by our group and others in hippocampus (Cheong et al., 2013; Nosarti et al., 2002), caudate nucleus (Abernethy et al., 2002; Nosarti et al., 2008), thalamus (Cheong et al., 2013; Gimenez et al., 2006a), corpus callosum (Narberhaus et al., 2008; Nosarti et al., 2004; Taylor et al., 2011) and cerebellum (Allin et al., 2001; Taylor et al., 2011). Using voxel based morphometry (VBM), we conducted the largest study to date which demonstrated widespread GM and white matter (WM) alterations especially in frontal and temporal lobes in mid-adolescence, which mediated cognitive impairment (Nosarti et al., 2008). Other studies in similar samples obtained consistent findings (Gimenez et al., 2006b; Nagy et al., 2009), whereas investigations on cortical morphology reported a thinner inferior frontal cortex in VPT adolescents vs. controls (Frye et al., 2010). Such findings could be interpreted within a ‘neuroplastic’ framework, which posits that developmental changes in any brain region may result in a cascade of alterations in many other regions (Hack and Taylor, 2000).

Despite strong evidence that neurodevelopmental anatomical alterations are present in VPT/VLBW children and adolescents, little is known about the nature and course of their brain development when they reach adulthood. Both increases and decreases in GM and WM volumes have been described in VPT/VLBW young adults compared to controls, especially in internal capsule, insula, prefrontal cortex, medial temporal/parahippocampal gyrus and putamen (Allin et al., 2004). Reductions in cortical surface area and cortical thickness alterations in prefrontal, temporal and parietal regions have also been found (Bjuland et al., 2013; Skranes et al., 2013). Finally, changes in WM microstructure, as assessed by diffusion tensor imaging, have been reported in several areas including the corpus callosum, corticospinal tracts, cortical association tracts, cerebellar penducle and corona radiata (Allin et al., 2011; Eikenes et al., 2011).

A question which remains unanswered is whether the structural brain differences observed between VPT individuals and controls at a given time point represent delays in the course of maturation (i.e., trajectory of brain development) or long-lasting brain alterations (Nosarti et al., 2008). The results of studies published to date suggest that such differences diminish with time. In the VPT/VLBW cohort from the University Hospital in Trondheim, Norway, cortical thickness deviations seemed to be more pronounced at age 15 (Martinussen et al., 2005) compared to age 20 (Bjuland et al., 2013), and we previously reported that the surface area of the corpus callosum did not differ between VPT born individuals at age 19 compared to controls, while it was significantly smaller in the VPT group when the same sample was studied in mid-adolescence (Allin et al., 2007).

It is important to study the association between structural brain alterations and cognitive/behavioural outcome measures (Allin et al., 2011; Bjuland et al., 2013; Eikenes et al., 2011; Skranes et al., 2013), as this could contribute to elucidate the causes underlying the increased risk in VPT/VLBW young adults of experiencing medical and social disabilities (Moster et al., 2008), executive function deficits (Nosarti et al., 2007), psychiatric disorder (Nosarti et al., 2012) and neurological abnormalities, even in the absence of neurodevelopmental impairments (Miskovic et al., 2009).

The current study aimed to assess regional GM and WM volumes in VPT-born young adults and controls using VBM. We hypothesized that VPT individuals would display WM and GM alterations predominantly in frontal, temporal, and occipital regions and cerebellum, as observed in a larger cohort in adolescence (Nosarti et al., 2008), but that by early adulthood these alterations would be less extensive than at previous assessment (Allin et al., 2007; Bjuland et al., 2013). We further hypothesized that GM and WM volumes in regions where significant between-group differences are observed would be associated with gestational age as well as with neurodevelopmental outcome (Cheong et al., 2013; Nosarti et al., 2008; Taylor et al., 2011).

## 2 Materials and methods

### 2.1 Study population

We studied a cohort of individuals who were born in 1983–84 before 33 weeks of gestation and admitted consecutively to the Neonatal Unit of University College London Hospital (UCLH). A total of 302 individuals were enrolled for follow-up (Allin et al., 2006). At 14–15 years, 90 individuals received a comprehensive cognitive and behavioural assessment and had an MRI scan (Nosarti et al., 2008). At age 19–20 years, 93 individuals were assessed and had an MRI scan. Seventy-four VPT individuals (82.2%) received an MRI scan at both time points. Results of diffusion tensor MRI analyses in the sample which forms the basis of the current study are reported elsewhere (Allin et al., 2011).

For the assessment at 14–15 years, 71 controls were recruited by advertisement in the local press (South London) and selected according to age and socio-demographic characteristics. At age 19–20 years 50 controls were assessed; 34 (47.9%) controls received an MRI scan at both time points. The remaining 19 controls were also recruited by advertisement in the local press and selected according to age and socio-demographic characteristics. Inclusion criteria were full-term birth (38–42 weeks) and birth weight >2500 g. Exclusion criteria were any history of neurological problems including meningitis, head injury and cerebral infections.

Reasons for attrition between follow-up studies included unavailability of current contact details, participants' unwillingness to participate in the study and contraindications for MRI including the presence of metallic implants.

Ethical approval for the study was obtained from the Institute of Psychiatry, King's College, London, Ethics Committee (Research). Written informed consent for the assessment, including MRI, was obtained from all participants.

### 2.2 Neurodevelopmental, behavioural outcome data and socio-economic status

The Wechsler Abbreviated Scale of Intelligence (WASI) (Wechsler, 1999) was used to provide estimates of full-scale IQ and comprised four scales: Vocabulary, Similarities, Matrix Reasoning and Block Design; the Visual Reproduction test of the Wechsler Memory Scale-Revised (WMS-R) (Wechsler, 1987), which assesses immediate and delayed recall of non-verbal material, was used to assess learning and memory; the Controlled Oral Word Association Test (COWAT) (Benton and Hamsher, 1976) (phonemic fluency) and the Hayling Sentence Completion Test (Burgess and Shallice, 1997) were used to assess executive function and in particular cognitive flexibility. A ‘global executive’ score was calculated as the sum of *Z* scores from the HSCT and the COWAT; and a ‘non-verbal memory’ score was calculated as the sum of *Z* scores from the WMS-R immediate and the WMS-R delayed tests. For VPT participants *Z* scores were obtained using means and SDs from controls, which by default were set at 0 and 1. The Clinical Interview Schedule-Revised (CIS-R) (Lewis et al., 1992) was used to measure the frequency and severity of non-psychotic psychiatric symptoms.

Socio-economic status (SES) was measured by Her Majesty's Stationary Office Standard Occupational Classification criteria (Her Majesty's Stationery Office (HMSO) 1991). The following SES bands were used: I−II = managerial and professional; III = intermediate (e.g., small employers and own account); IV−V = working (i.e., lower supervisory and technical, routine).

### 2.3 Analysis of neonatal, socio-demographic, cognitive and behavioural data

Data were analysed with IBM® SPSS® Statistics 21.0. Neonatal characteristics (gestational age, birth weight, neonatal ultrasound classification) and socio-demographic variables (age at assessment, sex and SES) were analysed with Chi-square tests or univariate analysis of variance, as applicable. 95% confidence intervals were calculated. Between-group differences in cognitive and behavioural measures were assessed by univariate analysis of covariance adjusting for age at assessment. Between-group differences in WASI sub-scores were analysed with multivariate analysis of covariance, using age at assessment as a confounder.

### 2.4 MRI data acquisition

Magnetic resonance imaging was performed using a 1.5 Tesla system (General Electric Medical Systems, Milwaukee, WI). Three-dimensional T1-weighted spoiled gradient-echo sequences with 124 1.5 mm slices (TR 35 ms, TEef 5 ms, flip angle 35°) were obtained.

### 2.5 MRI image analysis

All images were processed using VBM8 toolbox (http://dbm.neuro.uni-jena.de/vbm/) in SPM8 (http://www.fil.ion.ucl.ac.uk/spm/software/spm8/), running on Matlab Version 7.8.0.347 (MathWorks, Natick, USA). During VBM processing, all images were bias-corrected using a Bayesian framework to model intensity non-uniformity and a tissue probability map was overlaid onto each image. Each image was skull-stripped and segmented into GM and WM and affine-registered to Montreal Neurological Institute (MNI) space. Then, a template was created for GM and WM, respectively, using the mean of all segmented images as proposed by the DARTEL algorithm (Ashburner, 2007). Subsequently, deformation fields were calculated between each segmented image and the final DARTEL-created template. Warping was carried out modulating only for non-linear components, thus allowing comparison of the absolute amount of tissue class corrected for individual brain sizes. The resultant images were smoothed with a 12 mm full-width at half-maximum (FWHM) isotropic Gaussian kernel.

Age at assessment was controlled in all analyses. Group (VPT and control) and gender (male and female) were used as fixed factors. We compared the estimates of the adjusted group means (VPT versus control) firstly including and secondly excluding the VPT participants with evidence of periventricular haemorrhage (PVH) and ventricular dilatation (DIL) on neonatal ultrasound, employing a linear contrast, which computed *t* values at each voxel. This analysis was performed as we previously observed that very preterm-born individuals who experienced PVH and DIL exhibited the greatest WM and GM alterations (Nosarti et al., 2008). The ensuing statistical parametric maps (SPM) from these contrasts (built on probability assignment to GM or WM) were converted to the unit normal distribution SPM [Z]. Results tables report voxel level local maxima more than 8.0 mm apart with a *p* value corrected for family-wise error (FWE) of <0.05, and a conservative threshold on cluster size, comprising 50 or more contiguous significant voxels.

GM and WM eigenvalues for each cluster in each scan using SPM's volume of interest (VOI) data extraction tool were then computed from all clusters where significant group differences were found. One-sample Kolmogorov–Smirnov tests were performed, which revealed a normal distribution of the data. Cluster local maxima were converted from the standard MNI to Talairach coordinates using the Lancaster transform (i.e., icdbm2tal) function in in GingerALE Version 2.1.1 software (Laird et al., 2010) in order to label them using the Co-Planar Stereotaxic Atlas of the Human Brain (Talairach and Tournoux, 1988).

### 2.6 GM, WM volumes and gestational age

The association between GM and WM volumes and gestational age was investigated with stepwise regression models in the VPT group. Gestational age was used as dependent variable, and eigenvalues extracted from all the regions where between group GM and WM volume differences were observed were used as predictors.

### 2.7 GM, WM volumes and cognitive data

The quantitative contribution of regional brain alterations in GM and WM volumes to cognitive outcome was investigated with linear regression models. The neurodevelopmental scores where VPT individuals showed significantly lower scores from controls, full-scale IQ, global executive function and non-verbal memory total score were used as dependent variables, and eigenvalues extracted from the regions of between group GM and WM differences and group (VPT or control) as predictors. The correlations between GM and WM volumes and cognitive outcomes were further investigated separately for the VPT and control groups. Finally, we categorized all participants into ‘cognitively impaired’ or ‘cognitively unimpaired’. Participants scoring one or more standard deviations below the mean of the control group for both global executive function and memory total scores were regarded as ‘cognitively impaired’, following definitions commonly used in neuropsychological research (Lezak, 1995). In order to study whether structural GM and WM alterations between the ‘cognitively impaired’ and ‘cognitively unimpaired’ groups were specific to areas showing differences between VPT individuals and controls, we carried out a voxel-based analysis similar to the one excluding the PVH + DIL group, in this case excluding the ‘cognitively unimpaired’ individuals in both VPT and control groups. Analyses comparing GM and WM volumes in ‘cognitively unimpaired’ individuals only (in both the VPT and control groups) were also performed.

## 3 Results

The 93 VPT individuals assessed at age 19–20 did not differ from the 302 individuals who were enrolled for follow-up in terms of gestational age (*F*(301) = 2.4, *p* > 0.05), birth weight (*F*(301) = 0.8, *p* > 0.05) and Apgar score at 1 and 5 min (*F*(301) = 0.005, *p* > 0.05 and *F*(301) = 0.03, *p* > 0.05, respectively).

From a total of 143 brain images, 111 were included in this study (43 controls, 68 VPT). Thirty-two participants' scans (7 controls, 25 VPT) were excluded from the analyses after performing a sample homogeneity check and visual inspection. The exclusion criteria were (1) the largest squared distance to mean GM/WM values of the whole sample; and (2) acquisition artefacts (e.g., motion, aliasing or ‘clipping’ of frontal/occipital lobe) or ventricular enlargement. Participants in the current analysis did not differ from non-participants in gestational age (*F*(142) = 3.3, *p* = 0.07) (although there was a trend for non-participants to have a younger gestational age than non-participants), birth weight (*F*(142) = 1.1, *p* = 0.29) and full-scale IQ at assessment (*F*(142) = 0.007, *p* = 0.93).

Participants' neonatal and socio-demographic details are given in Table 1. VPT-born participants statistically differed from controls in terms of age at assessment, controls being slightly younger.

VPT- born young adults had lower IQ compared to controls, and they displayed worse performance on global scores of executive function and non-verbal memory (see Table 2). Executive function scores were not significantly associated with length of gestation in the preterm group (*r* = 0.07, *p* = 0.55), while non-verbal memory scores were positively associated with gestational age (*r* = 0.31, *p* = 0.009). Correlations among all the cognitive measures, separately for the VPT and the control groups, are reported in Supplementary Table 1.

### 3.1 GM and WM volume differences

**Fig. 1:**
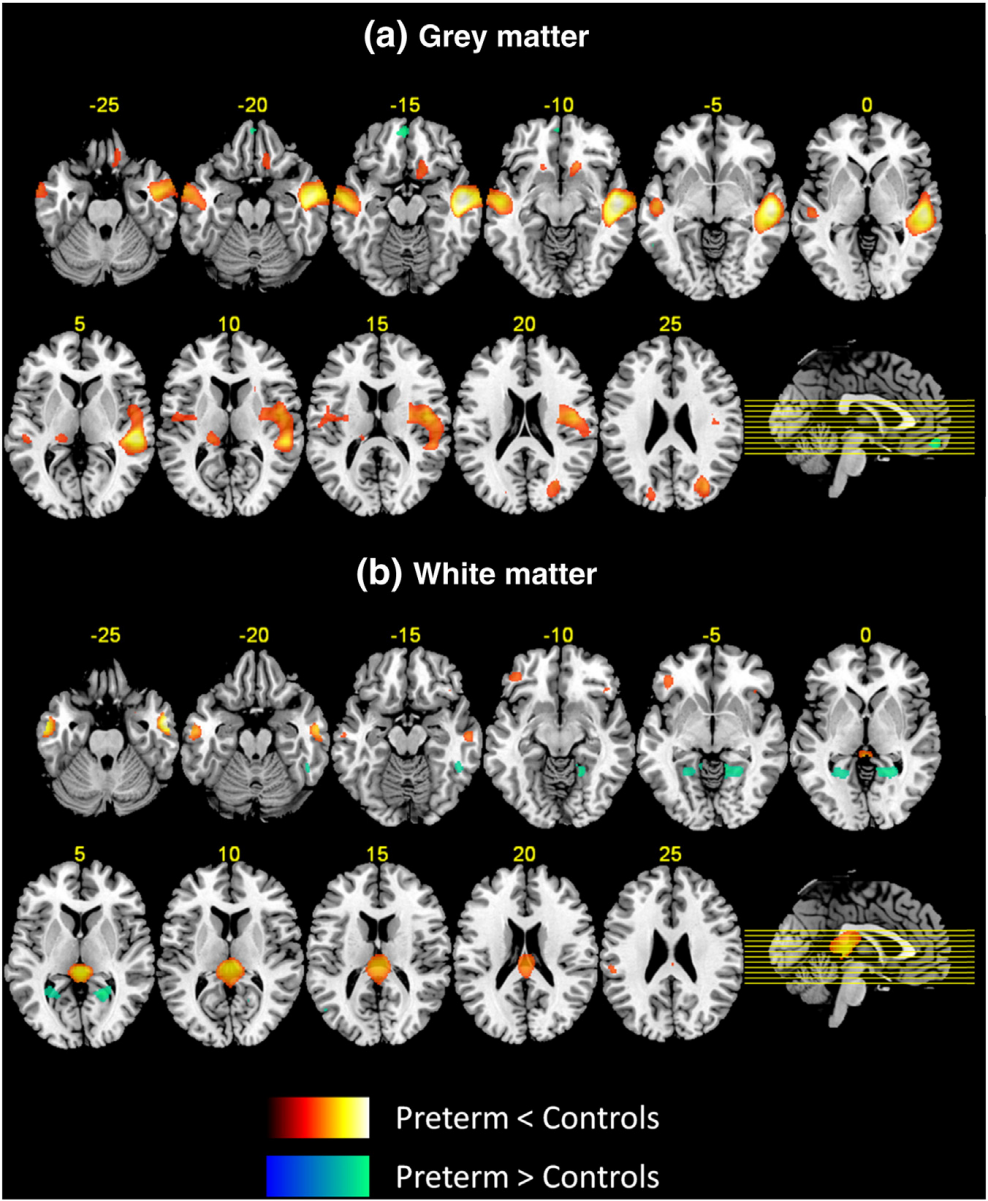
Three-dimensional representation of areas of decreased and increased GM (a) and WM (b) volumes in VPT individuals compared to controls. All areas displayed were significant at *p* < 0.05 FWE corrected. Lighter colour refers to higher T peak-level statistics.

Between group differences in GM volume were identified in large clusters where VPT individuals displayed smaller volumes (e.g., the absolute amount of regional GM) compared to controls. These were mainly bilaterally distributed and included the temporal lobes, right insula and medial frontal gyrus, left anterior cingulate gyrus and medial occipital lobe. Subcortical GM areas such as the thalamus and the caudate nucleus were also reduced in volume in the VPT group. On the other hand, larger GM volume in the VPT group was only observed in one cluster with local maxima in the medial/anterior frontal cortex (Table 3, Fig. 1a).

When the PVH + DIL group was excluded from the analyses, VPT individuals showed smaller GM volume in brain regions almost identical in number and location to those observed in the analysis comparing all VPT individuals to controls (see Supplementary Table 2), with the exception of two clusters in Brodmann area (BA) 25/24, one centred in right medial frontal gyrus (9, 26, −14) and the other centred in left cingulate gyrus (−16, 19, −4), which were no longer statistically significant. When the PVH + DIL group was excluded, the only areas displaying larger GM volume in VPT individuals compared to controls remained centred in medial frontal gyrus (BA 10), but extended to cingulate gyrus (BA 24).

Between group differences in WM volume were identified in large clusters where VPT individuals displayed smaller volumes (e.g., the absolute amount of regional WM) compared to controls. Smaller subcortical WM volume in the VPT group was concentrated bilaterally in temporal and frontal regions, posterior corpus callosum extending to thalamus and hippocampal fornix and left inferior parietal lobule. Larger WM volume was found predominantly in right posterior brain regions, in lingual, parahippocampal, fusiform gyri and cerebellum (Table 3, Fig. 1b).

When the PVH + DIL group was excluded from the analyses, again VPT individuals showed clusters of smaller subcortical WM volume compared to controls in similar locations to those obtained in the analysis comparing all VPT individuals to controls, although their number was significantly reduced (see Supplementary Table 2). When investigating larger subcortical WM volume in the VPT sample excluding the PVH + DIL group, all the regions which were statistically significant when considering the whole VPT group remained significant, as well as three more clusters centred in left anterior cerebellum, in areas beneath the left parahippocampal and right medial frontal gyri.

Significant main effects for gender (males versus females) were observed for GM volume, males having larger volume in right cuneus (BA 18) (Talairach coordinates: 0, −96, 10; *Z* = 5.80) and left putamen (−14, 14, −8; *Z* = 4.54). Females had larger subcortical WM volumes than males in an area beneath the right postcentral gyrus (52, −25, 40; *Z* = 4.97) and in right posterior cerebellum (13, −63, −31; *Z* = 4.54). Results of interaction analyses investigating group (VPT versus control) by gender (males versus females) were not statistically significant.

### 3.2 Gestational age and GM and WM volumes in VPT individuals

Stepwise regression analyses revealed that gestational age was positively correlated with GM and WM volumes in right medial temporal gyrus and with subcortical WM volume in an area beneath the left inferior parietal lobule, regions where VPT individuals demonstrated decreased volume compared to controls. Gestational age was negatively associated with subcortical WM volume in areas beneath the left lingual and right fusiform gyri, regions where VPT individuals demonstrated increased volume compared to controls (*R*^2^ = 0.47; *F* = 11.14, *p* < 0.0001) (see Table 4).

### 3.3 Cognitive outcome and GM and WM volumes

Results of stepwise linear regression analyses revealed that structural alterations in subcortical WM in areas beneath the left inferior frontal gyrus (where VPT participants showed smaller volumes than controls) (Talairach coordinates: −40, 35, 0; *R*^2^ change = 0.14) and the right lingual gyrus (where VPT participants showed larger volumes than controls) (19, −51, 3; *R*^2^ change = 0.06) accounted for 18% of the variance of full-scale IQ (*F* = 12.9, *p* < 0.0001). Group was not statistically significant (β = −0.18, *p* > 0.05).

Results further revealed that structural alterations in WM in posterior corpus callosum/thalamus/fornix (Talairach coordinates: −1, −31, 5; *R*^2^ change = 0.09), and GM in left (−54, −10, −8; *R*^2^ change = 0.08) and right medial temporal gyri (29, −72, 20; *R*^2^ change = 0.04) (all areas where VPT participants showed smaller volumes than controls) accounted for 21% of the variance of executive function (*F* = 9.9, *p* < 0.0001) and WM in posterior corpus callosum/thalamus/fornix (−1, −31, 5) alone accounted for 17% of the variance of total memory scores (*F* = 9.9, *p* < 0.0001), while group was not statistically significant (β = 0.04, *p* > 0.05 and β = −0.05, *p* > 0.05, respectively).

**Fig. 2:**
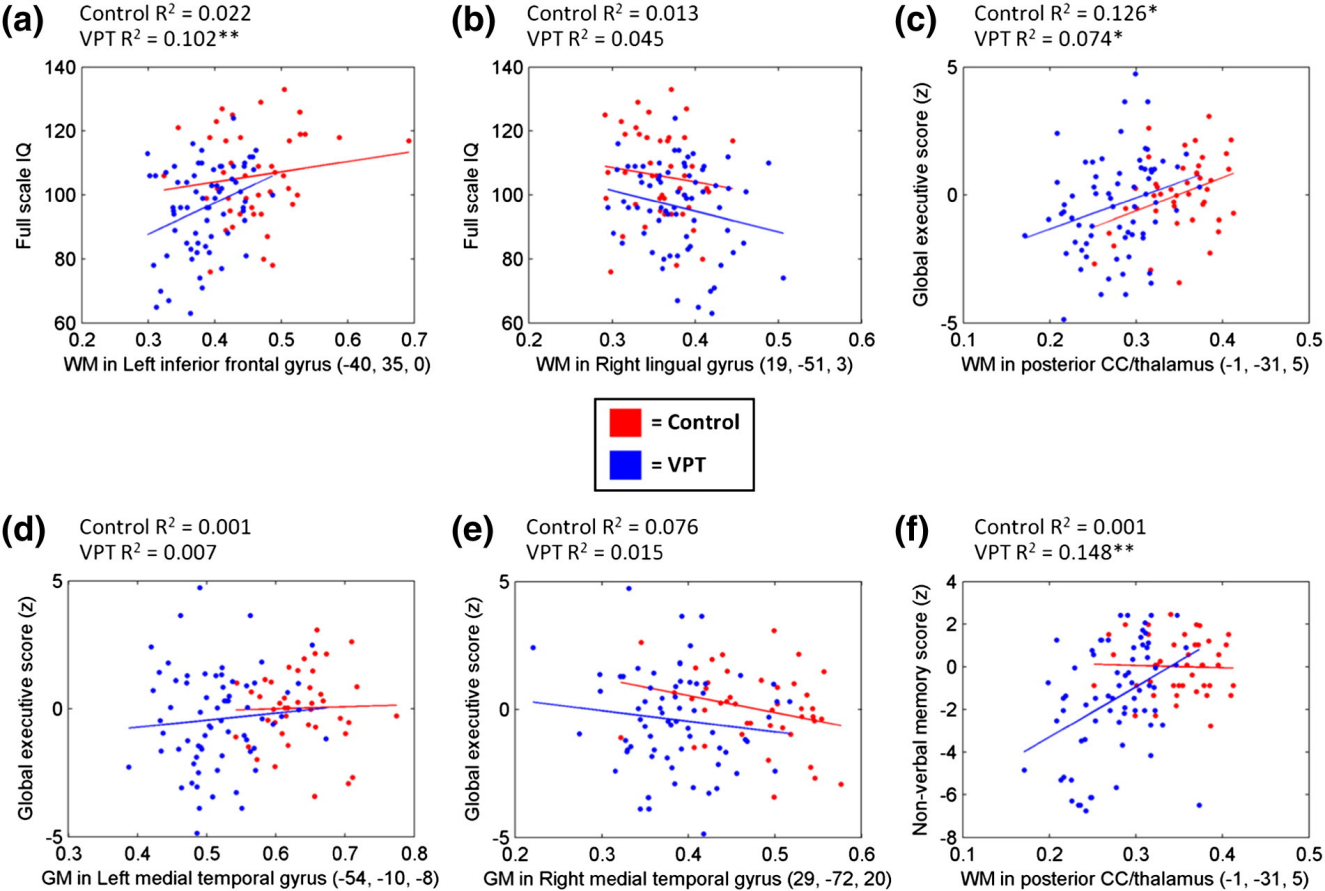
Correlation between eigenvalues extracted from the brain regions which were significantly associated with cognitive outcome measures, by group.

The correlation between eigenvalues extracted from the brain regions which were significantly associated with our outcome measures, by group, is shown in Fig. 2. As displayed in Fig. 2a, there was an interaction between group and eigenvalues extracted from WM in left inferior frontal gyrus and full-scale IQ; larger subcortical WM volume in an area beneath the left inferior frontal gyrus correlated with higher IQ scores in VPT individuals, whereas this association was weak in controls. A significant interaction was further observed between group and eigenvalues extracted from WM in posterior corpus callosum/thalamus/fornix and non-verbal memory scores (Fig. 2f). Larger WM volume in posterior corpus callosum/thalamus/fornix correlated with higher non-verbal memory scores in VPT individuals, whereas such association was not noted in controls.

All other correlation results showed similar patterns of association between regional volumetric measures and cognitive scores in the VPT and control groups (Fig. 2b, c, d, e).

25% of VPT participants were categorized as ‘cognitively impaired’ on the basis of their mean performance on both global executive function and total memory scores, compared to only one control (2.3%) (χ^2^ = 9.97, *p* = 0.001). Results of VBM analyses considering only the ‘cognitively impaired’ VPT sample compared to controls found that the areas which statistically differed between groups were centred in the exact locations as those observed in all VPT participants (100% of the sample, ‘cognitively impaired’ plus ‘cognitively unimpaired’) vs. controls, although the number of regions of differences were fewer than those observed in the analyses considering all VPT individuals vs. controls. These are listed in Table 5.

VBM analyses considering only the ‘cognitively unimpaired’ VPT-born individuals and controls found very similar GM and WM volume differences between the groups to those observed in the analyses considering all VPT individuals vs. controls. These are shown in Supplementary Table 3.

## 4 Discussion

The results of this study suggest that extensive structural brain alterations are present in early adulthood in VPT individuals, and they mediate high-order cognitive functions. We previously hypothesized that GM and WM volumetric differences observed in a VPT sample in mid-adolescence may have represented developmental delays, and that the GM and WM maturational patterns observed in our sample were consistent with the age-related stages typically observed in younger subjects (Nosarti et al., 2008). The current results, however, show only partial evidence that VPT individuals ‘catch up’ with controls by age 20, suggesting the trajectory of brain development following VPT birth may not only be delayed, but also fundamentally distinctive.

As observed in a larger VPT cohort at mid-adolescence (Nosarti et al., 2008), both smaller and larger GM and WM volumes were found in VPT individuals compared to controls, although regional volumetric alterations were more circumscribed compared to previous findings, in line with other studies investigating cortical thickness in similar samples (Bjuland et al., 2013; Martinussen et al., 2005).

### 4.1 GM volume

Only very few studies to date have investigated structural brain changes in VPT/VLBW samples beyond adolescence, and have also reported structural alterations in the areas of reduced GM volume observed here for the VPT group. For instance, reduction in cortical surface area and cortical thickness in VPT/VLBW samples compared to controls was observed in temporal regions (Bjuland et al., 2013; Skranes et al., 2013) and decreased GM volume in insula and putamen (Allin et al., 2004). In a larger VPT-born sample than the one currently investigated, aged 15 years, we previously reported GM volume decreases in temporal, frontal, and occipital cortices, including insula, cuneus, thalamus, caudate nucleus and putamen (Nosarti et al., 2008). Age-related increases in medial temporal lobe have been described up to puberty (Hu et al., 2013), thalamic volumes have been documented as significantly reducing with age (from 7 to 16 years) (Sowell et al., 2002) and caudate nucleus has been described as following a U-shape developmental trajectory, peaking in childhood (from 7.5 to 10 years) (Lenroot and Giedd, 2006). We therefore hypothesize that decreased GM volume in these regions in VPT individuals in their 20s is likely to reflect permanent structural alterations.

Temporal regions, thalamus, caudate nucleus and putamen may be especially vulnerable to damage during the third trimester of gestation, during which several developmental events occur, involving premyelinating oligodendrocytes (pre-OLs), axons, microglia, subplate neurons and cell migration from subventricular zone (Volpe, 2009). Alterations in GM volume in these areas may further represent secondary effects of WM injury, which has been associated with deep GM growth failure resulting in focal tissue loss (Boardman et al., 2010; Inder et al., 2005; Miller and Ferriero, 2009). A possible mechanism underlying the observed GM deficits in the VPT-born sample at this specific stage of development could be exaggerated synaptic pruning (i.e., ‘hyperpruning’), which is believed to generate a refinement in interneuron function and connectivity, and happens from adolescence through early adulthood (Gogtay et al., 2004).

Maturational changes in occipital lobes have been described as showing maximum development at around 20 years, and in frontal lobes up to the third decade of life (Westlye et al., 2010), therefore it remains to be ascertained whether altered patterns of GM development following VPT birth in frontal and occipital regions reflect an age-specific endophenotype. This hypothesis has been suggested in healthy siblings of individuals with schizophrenia, who share regional GM alterations with their probands in childhood, but show compensatory normalization by early adulthood (Gogtay et al., 2007).

At current assessment, the VPT group displayed increased GM volume compared to controls only in one large cluster in medial/anterior frontal cortex, while in mid-adolescence numerous areas of increased GM volume were found in a larger VPT sample, including frontal and temporal cortices and the cerebellum (Nosarti et al., 2008). It is of interest to note that thicker cortex in medial/anterior frontal regions bilaterally was also recently reported by Bjuland and colleagues (2013) in a VLBW/VTP adult sample. These findings may represent delayed processes of synaptic pruning, as regressive events occur in frontal lobes into the 30s (Westlye et al., 2010); alternatively, they may reflect a region-specific failure of synaptic pruning (Keshavan et al., 1994).

When the PVH + DIL group was excluded from the analyses, VPT individuals showed fewer areas of smaller GM volume compared to controls, centred in the same location as those observed in the analysis including the whole VPT group. These analyses suggest that severe perinatal brain injury represents an additional risk factor for long-term widespread anatomical alterations in addition to the effects of VPT birth (Nosarti et al., 2008), possibly reflecting a biological risk underlying the impaired cognitive and behavioural profiles often described in sub-samples of VPT survivors who experienced early brain damage (Nosarti et al., 2011; Sherlock et al., 2005).

### 4.2 WM volume

Smaller WM volume in VPT individuals were concentrated in temporal and inferior frontal regions bilaterally, posterior corpus callosum extending to thalamus/hippocampal fornix and left inferior parietal gyrus. These results are quite different from those observed in mid-adolescence in a larger VPT-born sample, which included more numerous areas such as the brainstem, internal capsule, subthalamic nuclei, as well as occipito-frontal fasciculus. However, WM volume decrements in temporal, frontal regions and insula were also observed in mid-adolescence (Nosarti et al., 2008). The prevalence of WM injury following VPT birth may be related to the developmental vulnerability of glial precursor cells before 33 gestational weeks (Back et al., 2007), which are implicated in the generation of oligodendroglia, that play a critical role in myelination and the formation of guidance molecules (Weiss et al., 2004). Supporting evidence for this hypothesis is given by the observation of WM alterations in VPT infants at term equivalent among some of the earliest regions of the brain to myelinate (Counsell et al., 2002), including the thalamus (Ball et al., 2012) and cerebellum (Srinivasan et al., 2006). Results of complementary studies using diffusion MRI have described WM microstructural alterations, predominantly decreased fractional anisotropy (FA), in VPT adolescents (Allin et al., 2011; Eikenes et al., 2011; Salvan et al., 2013).

Recent investigations have shown that WM linearly increases throughout the first four decades of life, possibly due to ongoing myelin maturation, and especially in later-myelinating prefrontal association regions (Bartzokis et al., 2010). It therefore remains to be ascertained whether the WM volumetric alterations in this VPT sample represent a delayed shift of normal maturational processes.

Larger WM volume in the VPT-born group was found predominantly in right posterior brain regions, in areas beneath the lingual, parahippocampal, fusiform gyri and in cerebellum. Again, these findings are less extensive than those observed in mid-adolescence, which reported WM excesses in temporal, parietal, frontal regions, and in an area beneath the fusiform gyrus (Nosarti et al., 2008). Larger WM volumes in VPT individuals may represent extensive compensatory processes following cell death accompanying intraventricular haemorrhage and ventriculomegaly and disruption of subsequent WM development (Kuban et al., 1999), thus reflecting both destructive and adaptive developmental processes. Larger regional WM volume has been described in disorders postulating disturbances in neurodevelopment, such as schizophrenia (Lee et al., 2007), developmental language disorder and autism (Mostofsky et al., 2007).

When the PVH + DIL group was excluded from the analyses, the number of clusters showing smaller subcortical WM volume in VPT individuals compared to controls substantially decreased, while the number of clusters showing larger subcortical WM volume in the VPT group substantially increased. These results suggest that the PVH + DIL group experienced the greatest alterations in WM, consistent with the results of our adolescent study (Nosarti et al., 2008). In terms of WM microstructure, increased FA in association with more severe neonatal brain injury has been observed in VPT samples (Allin et al., 2011; Myall et al., 2013), which may underlie increased axonal packing density and axonal straightening secondary to ventricular enlargement (Myall et al., 2013).

#### 4.3 GM and WM volume and gender

Our results highlighted significant main effects for gender, with males displaying larger GM volume than females in right cuneus and left putamen, and females demonstrating larger WM volumes than males in an area beneath the right postcentral gyrus and in right posterior cerebellum. Sexual dimorphism in the human brain has been extensively studied (Giedd et al., 2012), and some of the current findings are in line with the results of previous investigations, which described lager volume in bilateral putamen in adult males (Tang et al., 2013). Not entirely consistent with our findings of increased regional WM volume in posterior cerebellum in females, whole-cerebellar volume has been reported as being 10–13% larger in males (Tiemeier et al., 2010). However, it has been suggested that differences in regional volumes between males and females may reflect the effects of differences in brain size (approximately 10% larger in males) rather than characteristic cytoarchitecture (Brun et al., 2009). Different neuroimaging methods, inclusion of possible confounders and subjects' age at scan may prevent a direct comparison of study findings.

Results of interaction analyses investigating group by gender were not statistically significant. Other studies have revealed gender-specific structural brain differences in VPT individuals compared to controls in corpus callosum and interior capsule (Rose et al., 2009) and in cerebral WM volume (Constable et al., 2008). In a younger sample of VPT participants, we previously reported a significant structure–function interaction between group and gender in respect to spelling abilities and GM volume in left frontal gyrus (Scott et al., 2011). Again, methodological differences between the studies may account for inconsistent findings.

### 4.4 GM and WM volume and gestational age

When investigating associations between GM and WM volumetric alterations in regions which differed between the groups and gestational age, the volume of five brain areas accounted for just under 50% of the model variance; these were GM in right medial temporal gyrus, WM in right medial temporal, inferior parietal, left lingual and right fusiform areas. A developmental window of vulnerability for WM injury is the third trimester of gestation, with the main potential target being pre-OLs (Volpe, 2009), therefore significant associations between gestational age and regional WM volumes could be interpreted in the context of the timing of such crucial maturational events, leading to subsequent disturbances in neurodevelopment (both volume decreases and increases) (Allin et al., 2004; Mostofsky et al., 2007; Nosarti et al., 2008).

### 4.5 GM and WM volumes and cognitive outcome

VPT individuals attained lower scores than controls on measures of general intelligence, executive function and non-verbal memory, in line with previous findings (Heinonen et al., 2013; Nosarti et al., 2007; Skranes et al., 2013). Full-scale IQ scores were significantly associated with subcortical WM volume in an area beneath the left inferior frontal gyrus (VPT > controls), consistent with findings in normative samples (Haier et al., 2004) and with the findings of Skranes and colleagues (2013) who found a significant association between IQ scores and surface area reduction in inferior frontal gyri in a VPT/ VLBW sample of a similar age to the one we studied. However, this association was statistically significant in VPT individuals and weak in controls. These results indicate that the more similar the WM matter volume to controls' values, the better the cognitive outcome in VPT individuals. These results are in line with our previous adolescent findings, which demonstrated that every 25% difference in regional GM and WM volumes in the VPT group compared to controls was associated with a significant increased risk of cognitive impairment (Nosarti et al., 2008).

Our results further indicate that among all cortical and subcortical regions where GM and WM alterations were noted in the VPT sample, WM reduction in posterior corpus callosum/thalamus/fornix contributed most significantly to cognitive outcome. The corpus callosum is the main route for inter-hemispheric connectivity, with fibres linking temporal–parietal–occipital regions passing through its posterior segment (de Lacoste et al., 1985). Associations between the surface area of the corpus callosum and executive function have been observed in VPT adolescents (Narberhaus et al., 2008). The posterior corpus callosum (e.g., splenium) has been specifically associated with language functions in VPT adolescents (Narberhaus et al., 2008; Northam et al., 2012), including verbal fluency (Nosarti et al., 2004), which contributed to make up the executive function score used here, as well as with visuospatial working memory in paediatric clinical samples (Treble et al., 2013).

The WM cluster centred in posterior corpus callosum extended to the thalamus, a region which due to its extensive reciprocal connections to most cortical regions, has been centrally implicated in tasks requiring executive control (Marzinzik et al., 2008) and non-verbal memory (Johnson and Ojemann, 2000). The thalamus has been described as being adversely affected by VPT birth at different ages (Ball et al., 2012; Cheong et al., 2013; Nosarti et al., 2008), together with its cortical connections to the prefrontal cortex, supplementary motor areas, occipital and temporal lobes (Ball et al., 2013).

The WM cluster centred in posterior corpus callosum/thalamus included the hippocampal fornix, a main connection of the hippocampal–diencephalic and parahippocampal–retrosplenial networks (Catani et al., 2013), which have been associated with memory and spatial orientation, respectively (Aggleton, 2008; Vann et al., 2009). Alterations of fornix WM microstructure assessed by diffusion MRI techniques have been described in a VPT sample of a similar age to the one studied here (Salvan et al., 2013) and have further been used as predictors of memory decline and progression to Alzheimer's disease (Mielke et al., 2012). Our results showed that larger WM volume in posterior corpus callosum/thalamus/fornix correlated with higher non-verbal memory scores in VPT individuals only, leading us to speculate that WM in this cluster (and especially in the hippocampal fornix) may be highly specialized, thus centrally implicated in non-verbal memory processing, so that the anatomical alterations observed in VPT samples have significant functional correlates. On the other hand, executive functions, which have been described to mature in parallel with the maturation of the prefrontal cortex (Luciana et al., 2003), may be subserved by domain-general frontal brain networks, and thus would be less functionally affected by subtle alterations in other key regions of the ‘executive’ network.

In terms of GM volume, the clusters labelled as ‘medial temporal gyrus’ bilaterally, which were significantly associated with executive function scores, were very large and encompassed the parahippocampal region, including the parahippocampal, entorhinal, and perirhinal cortices (but not the hippocampus proper). These areas are highly interconnected and act as intersections between the hippocampus and several multimodal association regions of the brain including the parietal, temporal, and prefrontal cortices (Canto et al., 2008; Suzuki, 2009). The volume of the parahippocampal gyrus has been associated with both executive functions and non-verbal memory (Antonova et al., 2004), and more generally with complex cognitive operations involving processing of contextual information (Aminoff et al., 2013). In a previous study in a comparable sample, Skranes et al. (2012) reported that reduced cortical thickness in entorhinal cortex in VPT/VLBW adolescents correlated with low performance on executive function tests.

Skranes et al. (2012) hypothesized that thinning of the entorhinal cortex may have been secondary to WM injury, consistent with findings of a recent study which showed that VPT children with periventricular leukomalacia, compared to ‘control’ children born VPT, displayed GM volume decrements in medial temporal lobe and thalami (Zubiaurre-Elorza et al., 2011). The association between reduced WM volume in posterior corpus callosum/thalamus, reduced GM volume in medial temporal lobes and cognitive outcome observed here supports the hypothesis of a possible association between WM injury and alterations in GM, although further studies are needed to directly explore this assumption.

When GM and WM volumes were compared between ‘cognitively impaired’ VPT-born individuals and controls, the areas which statistically differed between the groups were centred in the exact locations as those observed when analysing the whole VPT group, although they were fewer. This may be due to a loss of statistical power, as the sample size included only one fourth of the original VPT sample. In fact analyses considering only ‘cognitively unimpaired’ study participants found very similar GM and WM volume differences between the groups to those observed in the whole group analyses, as they included 75% of the VPT subjects and all controls except one. These results suggest that the brain areas associated with cognitive impairment are the same that are vulnerable to anatomical alterations following VPT birth. Other studies have reported that GM alterations associated with specific psychiatric disorders (i.e., decreased GM volume in dorsolateral prefrontal cortex in first episode psychosis) were also directly associated with cognitive outcomes in the clinical group (Minatogawa-Chang et al., 2009), possibly underlying an intermediate phenotype. Taken together, as found in adolescence, our current results suggest that structural brain changes associated with VPT birth mediate the relationship with adult cognitive outcome (Nosarti et al., 2008).

### 4.6 Strengths and limitations

Strengths of this study include the use of SPM's DARTEL toolbox for registration of the MRI images, which in combination with VBM has been found to deliver the most consistently high registration accuracy compared to conventional VBM methods (Klein et al., 2009). A limitation of this study is the number of participants who could not be included in the final data analyses, which amount to just under a third (27%) of all VPT cases. Since ventricular enlargement was one of our exclusion criteria and since there also was a trend towards younger gestational age among non-participants, we may have been unable to document a possible brain pathology that would have increased the differences between VPT individuals and controls. Another limitation of our study is the age difference between the VPT and the control group, although the results presented were adjusted for age at assessment. A further limitation is the inclusion of term-born controls from the general population, which could differ from our VPT group in variables that have not been measured, although they were similar to VPT born individuals in gender distribution and socio-economic status. Finally, two clusters listed in Table 2, in which significant between-group differences were found, could not be visually displayed. The caudate head is not visible in Fig. 1(a) at *Z* = –5, whereas the putamen is visible at *Z* = –10. MNI coordinates for this cluster (16, 21, −12) were converted to Talairach space using the Lancaster transform function in GingerALE software, as described in Materials and methods section. Disagreement between the Talairach atlas and the MNI space is a widely recognized problem, especially for deep brain regions (Carmack et al., 2004). A similar issue concerns the display of the cluster centred in the anterior cerebellum, which is not visible in Fig. 1(b) at *Z* = –10 (but a small cerebellar cluster is visible at *Z* = –5). A more precise identification of cerebellar topography could be obtained with specific cerebellar atlases (Schmahmann et al., 1999).

## 5 Summary

VPT-born young adults continue to display alterations of GM and WM volumes into young adulthood, especially in temporal, frontal, parietal and occipital lobes and subcortical regions including the thalamus, caudate nucleus and putamen, although such alterations are more circumscribed compared to findings at younger ages. Volumetric differences in several brain areas are linearly associated with length of gestation and mediate cognitive outcome. Nevertheless, it still remains to be seen whether these changes reflect neurodevelopmental delays or long lasting structural alterations due to prematurity.

## Funding

The Wellcome Trust (grant 065699/Z/01/Z) funded this research.

## Figures and Tables

**Table 1:** Participants' neonatal and socio-demographic details.

	Control (n = 43)	VPT (n = 68)	Statistics
Gestational age at birth (weeks)	40.2 (39.5–40.9)	28.9 (28.4–29.3)	*F*(110) = 909.3, *p* < 0.0001
Birth weight (grams)	3329.7 (3197.8–3461.6)	1225.1 (1133.2–1317.0)	*F*(110) = 675.0, *p* < 0.0001
PVH + DIL (n, %)	n/a	29 (42.6%)	
Age at assessment	19.3 (19.0–19.7)	20.2 (19.9–20.5)	*F*(110) = 12.5, *p* = 0.001
Male / female (n)	23/20	32/36	χ^2^ = 0.4, *p* = 0.51
SES at assessment (n, %)^a^			χ^2^ = 4.9, *p* = 0.18
I−II	23 (54.8%)	25 (37.9%)	
III	12 (28.6%)	28 (42.4%)	
IV−V	7 (16.7%)	10 (15.2%)	
Missing	0	3 (4.5%)	

Mean and 95% confidence intervals (95% CI) are given, unless otherwise specified. PVH + DIL = presence of periventricular haemorrhage and ventricular dilatation on neonatal ultrasound. Please see Nosarti et al. (2008) for exact definitions.

a SES = socio economic status.

**Table 2:** Participants' cognitive and behavioural outcome variables.

	Control (n = 43)	VPT (n = 68)	Statistics
WASI full-scale IQ	106.0 (14.6)	96.2 (16.7)	*F*(110) = 13.0, *p* < 0.0001
WASI vocabulary	51.4 (10.7)	46.8 (11.7)	*F*(110) = 4.2, *p* = 0.047
WASI similarities	51.8 (8.2)	45.3 (9.1)	*F*(110) = 16.7, *p* < 0.0001
WASI matrix reasoning	53.4 (8.1)	48.4 (11.0)	*F*(110) = 5.6, *p* = 0.02
WASI block design	56.5 (8.3)	49.9 (11.6)	*F*(110) = 13.6, *p* < 0.0001
HSCT	5.1 (1.5)	5.9 (2.0)	*F*(110) = 4.1, *p* = 0.04
COWAT	41.2 (11.4)	36.5 (10.0)	*F*(110) = 5.2, *p* = 0.02
Global executive score a		−0.4 (1.9)	*F*(110) = 6.6, *p* = 0.01
WMS-R immediate	11.4 (2.1)	9.8 (3.3)	*F*(110) = 8.0, *p* = 0.006
WMS-R delayed	10.6 (2.9)	8.4 (3.3)	*F*(110) = 12.6, *p* = 0.001
Non-verbal memory score^a^		−1.5 (2.6)	*F*(110) = 10.7, *p* = 0.001
CIS-R	3.6 (3.90)	5.2 (6.16)	*F*(110) = 1.9, *p* = 0.17

Mean and standard deviations (SDs) are given, unless otherwise specified. WASI = Wechsler Abbreviated Scale of Intelligence; HSCT = Hayling Sentence Completion Test; COWAT = Controlled Oral Word Association Test; WMS-R immediate = Visual Reproduction test of the Wechsler Memory Scale-Revised immediate recall; WMS-R delayed = Visual Reproduction test of the Wechsler Memory Scale-Revised delayed recall; CIS-R = Clinical Interview Schedule-Revised.

a These scores are calculated as the sum of domain-specific *Z* scores: for VPT participants these were calculated using means and SDs from controls, which are omitted from the table as by default they are set at 0 and 1.

**Table 3:** Cluster maxima a structural differences in GM and WM volumes between VPT individuals and controls.

		Talairach coordinates^b^			SPM [Z]
		X	Y	*Z*	
Grey matter control > preterm					
	Medial temporal gyrus (BA 21)	51	−12	−9	<8.00
	Medial temporal gyrus (BA 21)	42	−2	−20	6.52
	Insula (BA 13)/postcentral gyrus (BA 43)	41	−10	20	6.47
	Medial temporal gyrus (BA 21)	− 54	−10	−8	7.06
	Medial temporal gyrus (BA 19)	29	−72	20	5.65
	Caudate head ext. to putamen^c^	14	19	−5	5.65
	Medial frontal gyrus (BA 25)	− 9	26	−14	4.85
	Thalamus (pulvinar)	− 16	−31	10	5.15
	Medial occipital gyrus (BA 18)	− 18	−79	19	4.82
	Heschl gyrus (BA 41)	− 46	−11	14	4.81
	Cingulate gyrus (BA 24)	− 16	19	−4	4.79
Grey matter control < preterm					
	Medial/anterior frontal gyrus (BA 10)	− 3	50	−6	4.80
White matter control > preterm					
	Medial temporal gyrus	51	−2	−21	< 8.00
	Superior temporal gyrus	42	10	−26	4.62
	Inferior / medial temporal gyrus	− 53	−6	−21	6.85
	Posterior corpus callosum ext. to thalamus (pulvinar) and including hippocampal fornix	− 1	−31	5	6.79
	Inferior frontal gyrus	− 40	35	0	4.78
	Inferior parietal lobule	− 51	−33	25	4.65
	Inferior frontal gyrus /insula	40	23	0	4.57
White matter control < preterm					
	Lingual gyrus	19	−51	3	5.38
	Parahippocampal gyrus	10	−46	−3	5.10
	Anterior cerebellum^c^	18	−47	−10	5.04
	Fusiform gyrus	45	−42	−14	4.78
	Lingual gyrus	− 22	−49	−1	4.77

a Voxel level local maxima more than 8.0 mm apart with a *p* value corrected for family-wise error (FWE) of <0.05, and a conservative threshold on cluster size, comprising 50 or more contiguous significant voxels are reported.

b Talairach coordinates (*x*, *y*, *z*), where *x* = left(–) vs. right(+); *y* = anterior(+) vs. posterior(−) and *z* = ventral(+) vs. dorsal(−). 0 is regarded as being at the level of the anterior commissure.

c These clusters are not visible in Fig. 1. Please refer to Discussion for further comments on this point.

**Table 4:** Correlations between GM and WM volumes and gestational age in VPT individuals.

		Beta	*p*
Grey matter			
	Medial temporal gyrus (BA 21) (51, −12, −9)	0.20	0.046
White matter			
	Medial temporal gyrus (51, −2, −21)	0.48	<0.0001
	Inferior parietal lobule (−51, −33, 25)	0.25	0.012
	Lingual gyrus (−22, −49, −1)	−0.24	0.028
	Fusiform gyrus (45, −42, −14)	−0.23	0.029

**Table 5:** Cluster maxima^a^ structural differences in GM and WM volumes between VPT individuals categorized as ‘cognitively impaired’ and controls.

		Talairach coordinates^b^			SPM [Z]
		X	Y	*Z*	
Grey matter control > preterm					
	Superior/medial temporal gyrus (BA 22)	51	−12	−7	6.64
	Medial temporal gyrus (BA 21)	−54	−12	−9	5.91
Grey matter control < preterm					
	n/s				
White matter control > preterm					
	Medial temporal gyrus	51	−2	−21	7.05
	Posterior corpus callosum ext. to thalamus (pulvinar) and including hippocampal fornix	1	−31	5	6.49
	Inferior/medial temporal gyrus	−53	−5	−21	5.83
	Parahippocampal gyrus	23	3	−17	5.06
	Inferior frontal gyrus	−37	37	−1	4.47
White matter control < preterm					
	Parahippocampal gyrus	10	−49	−3	4.70
	Lingual gyrus	19	−51	3	4.69

a Voxel level local maxima more than 8.0 mm apart with a *p* value corrected for family-wise error (FWE) of <0.05, and a conservative threshold on cluster size, comprising 50 or more contiguous significant voxels are reported.

b Talairach coordinates (*x*, *y*, *z*), where *x* = left(–) vs. right(^+^); *y* = anterior(+) vs. posterior(−) and *z* = ventral(+) vs. dorsal(−). 0 is regarded as being at the level of the anterior commissure.
