# Supplementary material for: Preterm birth and structural brain alterations in early adulthood
Source: Neuroimage Clin. 2014 Aug 13;6:180–91. doi: 10.1016/j.nicl.2014.08.005 (PMC4215396; doi:10.1016/j.nicl.2014.08.005)
Supplement: Supplementary file 1 — Supplementary material [file mmc1.pdf]

Supplementary material

Table 1. Correlation between cognitive outcome variables, by group.

	WASI Voc	WASI Sim	WASI MR	WASI BD	HSCT	COWAT	WMS Imm
WASI Sim		1.00					
CONTROL	0.63 (<0.0001)						
VPT	0.83 (<0.0001)						
WASI MR			1.00				
CONTROL	0.59 (<0.0001)	0.47 (0.002)					
VPT	0.49 (<0.0001)	0.48 (<0.0001)					
WASI BD				1.00			
CONTROL	0.58 (<0.0001)	0.50 (0.001)	0.54 (<0.0001)				
VPT	0.35 (0.005)	0.40 (0.001)	0.52 (<0.0001)				
HSCT					1.00		
CONTROL	0.30 (0.06)	0.24 (0.14)	0.12 (0.48)	0.30 (0.06)			
VPT	0.22 (0.08)	0.26 (0.04)	0.21 (0.09)	0.21 (0.10)			
COWAT						1.00	
CONTROL	0.64 (<0.0001)	0.36 (0.02)	0.34 (0.04)	0.40 (0.01)	0.38 (0.015)		
VPT	0.44 (<0.0001)	0.52 (<0.0001)	0.48 (<0.0001)	0.24 (0.06)	0.42 (<0.0001)		
WMS Imm							1.00
CONTROL	0.34 (0.03)	0.34 (0.03)	0.44 (0.005)	0.31 (0.05)	-0.02 (0.90)	0.14 (0.40)	
VPT	0.38 (0.002)	0.53 (<0.0001)	0.53 (<0.0001)	0.49 (<0.0001)	0.13 (0.28)	0.36 (0.004)	
WMS Del							
CONTROL	0.26 (0.09)	0.21 (0.20)	0.39 (0.12)	0.32 (0.05)	0.13 (0.41)	0.26 (0.11)	0.83 (<0.0001)
VPT	0.32 (0.009)	0.49 (<0.0001)	0.56 (<0.0001)	0.55 (<0.0001)	0.12 (0.36)	0.41 (0.001)	0.87 (<0.0001)

WASI = Wechsler Abbreviated Scale of Intelligence; WASI Sim = WASI Similarities; WASI Voc = WASI Vocabulary; WASI MR = WASI Matrix Reasoning; WASI BD = WASI Block Design; HSCT = Hayling Sentence Completion Test; COWAT = Controlled Oral Word Association Test; WMS Imm = Visual Reproduction test of the Wechsler Memory Scale-Revised immediate recall; WMS Del = Visual Reproduction test of the Wechsler Memory Scale-Revised delayed recall

Table 2. Cluster maxima^ structural differences in GM and WM volume between VPT individuals excluding those with periventricular haemorrhage and ventricular dilatation and controls.

		Talairach Coordinates*			SPM[Z]
		X	Y	Z	
**GREY MATTER Control > Preterm**					
	Medial temporal gyrus (BA 21)	49	-13	-9	>8.00
	Medial temporal gyrus (BA 21)	42	-2	-19	5.88
	Insula (BA 13)/Postcetral Gyrus (BA 43)	41	-9	20	6.09
	Medial temporal gyrus (BA 21)	-55	-10	-8	6.36
	Medial Temporal Gyrus (BA 19)	28	-72	20	5.81
	Caudate Head Ext. to Putamen	13	19	-5	5.94
	Thalamus	-21	-28	8	4.87
	Medial Occipital Gyrus (BA 18)	-20	-79	19	4.60
	Heschl Gyrus (BA 41)	-47	-11	14	4.68
**GREY MATTER Control < Preterm**					
	Medial Frontal/Cingulate Gyrus (BA 10, 24)	-4	45	3	4.67
**WHITE MATTER Control > Preterm**					
	Superior Temporal Gyrus	42	-3	-8	7.56
	Medial Temporal Gyrus	-47	-5	-11	6.18
	Posterior corpus callosum Ext. to Thalamus	-3	-31	7	6.46
**WHITE MATTER Control < Preterm**					
	Lingual gyrus	19	-51	3	5.28
	Parahippocampal gyrus	10	-46	-3	5.11
	Anterior Cerebellum	18	-47	-10	5.04
	Fusiform Gyrus	45	-42	-15	4.82
	Lingual Gyrus	-21	-49	-3	4.76
	Anterior Cerebellum	-9	-40	-4	4.65
	Parahippocampal gyrus	-27	-49	3	4.60
	Medial Frontal Gyrus	31	50	13	4.69

^ Voxel level local maxima more than 8.0 mm apart with a p value corrected for family-wise error (FWE) of <0.05, and a conservative threshold on cluster size, comprising 50 or more contiguous significant voxels are reported.

Talairach coordinates (x, y, z), where x = left(-) vs. right(+); y = anterior(+) vs. posterior(-) and z = ventral(+) vs. dorsal(-). 0 is regarded as being at the level of the anterior commissure.

Table 3. Cluster maxima^ structural differences in GM and WM volume between ‘cognitively unimpaired’ VPT individuals and ‘cognitively unimpaired’ controls.

		Talairach Coordinates*			SPM[Z]
		X	Y	Z	
**GREY MATTER Control > Preterm**					
	Medial Temporal Gyrus (BA 21)	51	-10	-9	>8.00
	Medial Temporal Gyrus (BA 21)	-54	-10	-8	6.49
	Medial Temporal Gyrus (BA 19)	29	-72	20	5.83
	Caudate Head Ext. to Putamen	14	19	-5	5.47
	Thalamus (Pulvinar)	-18	-30	9	5.02
	Heschl Gyrus (BA 41)	-46	-9	14	4.77
	Cingulate Gyrus (BA 24)	-16	19	-5	4.52
	Middle Frontal Gyrus (BA 46)	36	14	18	4.48
	Medial Frontal Gyrus (BA 25)	-10	26	-12	4.41
	Medial Occipital Gyrus (BA 18)	-18	-77	18	4.36
**GREY MATTER Control < Preterm**					
	n/s				
**WHITE MATTER Control > Preterm**					
	Medial temporal gyrus	51	-2	-21	7.71
	Inferior / Medial Temporal Gyrus	-53	-6	-21	6.20
	Posterior corpus callosum Ext. to Thalamus (Pulvinar) and including hippocampal fornix	-1	-31	5	5.80
**WHITE MATTER Control < Preterm**					
	Lingual Gyrus	19	-51	3	4.64
	Fusiform Gyrus	45	-42	-14	4.35
	Posterior cerebellum	2	-60	-33	4.33

^ Voxel level local maxima more than 8.0 mm apart with a p value corrected for family-wise error (FWE) of <0.05, and a conservative threshold on cluster size, comprising 50 or more contiguous significant voxels are reported.

* Talairach coordinates (x, y, z), where x = left(-) vs. right(+); y = anterior(+) vs. posterior(-) and z = ventral(+) vs. dorsal(-). 0 is regarded as being at the level of the anterior commissure.
